# Endoscopic Biopsy of Intra- and Paraventricular Brain Lesions: Practical Advantages and Clinical Experience

**DOI:** 10.3390/medicina62020260

**Published:** 2026-01-26

**Authors:** Bojan Jelaca, Nebojsa Lasica, Milica Gledja, Veljko Pantelic, Jagos Golubovic, Djula Djilvesi

**Affiliations:** 1 Faculty of Medicine, University of Novi Sad, 21000 Novi Sad, Serbia; nebojsa.lasica@mf.uns.ac.rs (N.L.); milica.gledja@gmail.com (M.G.); vpantelic97@gmail.com (V.P.); djula.djilvesi@mf.uns.ac.rs (D.D.); 2 Clinic of Neurosurgery, University Clinical Center of Vojvodina, 21000 Novi Sad, Serbia

**Keywords:** neuroendoscopy, biopsy technique, brain tumors, intraventricular lesions

## Abstract

*Background and Objectives*: Endoscopic biopsy of brain lesions plays an important role in the management of intra- and periventricular lesions. While the diagnostic yield of this technique has been reported with varying success across studies, its outcome is likely influenced by specific technical nuances of the procedure. However, the relationship between these technical factors and diagnostic accuracy remains understudied in the current literature. We aim to describe the procedural rationale, key anatomical considerations, and technical nuances of the endoscopic biopsy of intra- and paraventricular brain lesions, comparing standard tissue forceps with a side-cutting biopsy needle technique. *Materials and Methods*: We conducted a ten-year single-center, retrospective study of patients who underwent endoscopic biopsy for intra- and paraventricular brain lesions between January 2014 and December 2024. Patients were divided based on the biopsy technique used: the first group of 11 patients was treated using a side-cutting biopsy needle from the center of the lesion, while the second group of five patients underwent tissue sampling with standard endoscopic tissue cup forceps. The study evaluates and compares both approaches in terms of safety and diagnostic accuracy. *Results*: Endoscopic visualization enabled direct assessment of the biopsy site in both groups. Histopathological diagnoses were successfully obtained in all cases with a side-cutting biopsy needle (11/11, 100.0%), and in almost all cases with the cup forceps technique (4/5, 80.0%). In patients with obstructive hydrocephalus, an endoscopic third ventriculostomy (ETV) was performed as the first and therapeutic step in all procedures and two patients required a shunt procedure. *Conclusions*: Endoscopic biopsies utilizing a side-cutting biopsy needle strategy offer a promising adjunctive approach for selected intra- and paraventricular brain lesions. This method allows for direct visualization of the intraventricular surface, while the use of a needle biopsy can enhance the likelihood of obtaining diagnostically representative tissue with a high degree of reliability.

## 1. Introduction

The para- and intraventricular regions are anatomical sites where a wide spectrum of pathologies may occur. Because of their deep localization within the brain and surroundings that include important vascular elements, these lesions are often difficult to access using open microsurgical approaches [1]. Since 1978, when Fukushima first described the neuroendoscopic approach to pineal region lesions, this procedure has become a viable option for the management of both para- and intraventricular tumors [1]. Over time, the range of indications has steadily expanded, with numerous studies highlighting its advantages over microsurgical and stereotactic techniques [2,3].

Several factors contribute to the preference for the endoscopic approach. First, endoscopic biopsy can be combined with procedures such as third ventriculostomy or pellucidotomy, making it the treatment of choice in patients requiring urgent management of obstructive hydrocephalus, which occurs in the majority of cases [4]. Second, the histopathological diversity of lesions arising from these regions is considerable, considering that intra- and paraventricular tumors represent only about 2% of primary central nervous system tumors in adults [5,6]. Tumors found in this area include germ cell tumors, astrocytoma, pineal tumors, lymphomas, non-astrocytic glial tumors, embryonal tumors, sellar tumors, metastases, ependymomas, meningiomas, etc. [7]. In many cases, complete surgical resection is neither feasible nor required; instead, endoscopic biopsy followed by adjuvant radiotherapy or chemotherapy is sufficient [8]. The reliability of endoscopic biopsy in diagnostics of intra- and paraventricular lesions is estimated at 69.9–100%, with the wide range resulting from different methodologies and the experience of research teams [9,10].

Moreover, endoscopic biopsy is generally considered safer than stereotactic biopsy due to the surgeon’s ability to achieve effective intraoperative hemostasis under direct visualization. In stereotactic procedures, the reported rate of asymptomatic hemorrhages ranges from 3% to 10%, while symptomatic intracranial hemorrhages occur in 1% to 4.9% of the cases [11,12,13]. However, despite its advantages for intraoperative bleeding control, the use of cup forceps during endoscopic biopsy in some cases carries a risk of hemorrhage [13,14]. Although multiple studies over the past 25 years have evaluated the benefits and limitations of neuroendoscopic procedures, detailed reports on intraoperative hemorrhage—its incidence, management strategies, and outcomes—remain scarce [13,15]. Recognizing that cup forceps may negatively affect bleeding risk, alternative methods for biopsy of intra- and periventricular lesions have recently emerged. These techniques aim to contain the hemorrhage within the tumor [13].

Additionally, as we previously mentioned, the available data is still heterogenous regarding the diagnostic yield of neuroendoscopic procedures with contemporary studies reporting a range of 69.9% to 100% for obtaining an adequate tissue sample for histological diagnosis [16,17]. The wide range results from the wide heterogeneity of the studied population and the applied methodology [9,10]. Still, the majority of the papers argue that there is a diagnostic difficulty in said procedure, most commonly caused by an insufficient amount of tissue collected during biopsy, inaccurate sampling place, and the use of coagulation prior to biopsy [10].

Considering all of the factors listed above, we can conclude that intra- and paraventricular tumors constitute a rare and diagnostically demanding subgroup of intracranial pathology, in which the balance between procedural safety and diagnostic yield is of particular importance. Owing to the anatomical constraints and the diversity of potential lesion characteristics, no single biopsy technique can be considered universally optimal. Rather, the selection of the appropriate instrument should reflect a thoughtful integration of radiological findings, tumor features, and surgical expertise. Against this background, the present study aims to assess the efficacy of the side-cutting biopsy needle in comparison with the conventional cup forceps approach in the biopsy of intra- and paraventricular tumors.

## 2. Materials and Methods

### 2.1. Patient Data

**Figure 1:**
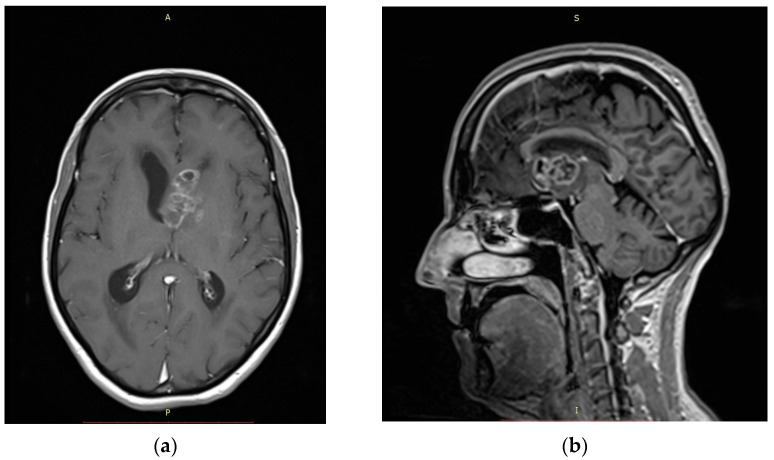
(**a**) Axial plane T1-weighted gadolinium-enhanced MRI scan of patient with glioblastoma located in the head of the caudate nucleus included in our study. (**b**) Sagittal plane T1-weighted gadolinium-enhanced MRI scan of the same patient.

**Figure 2:**
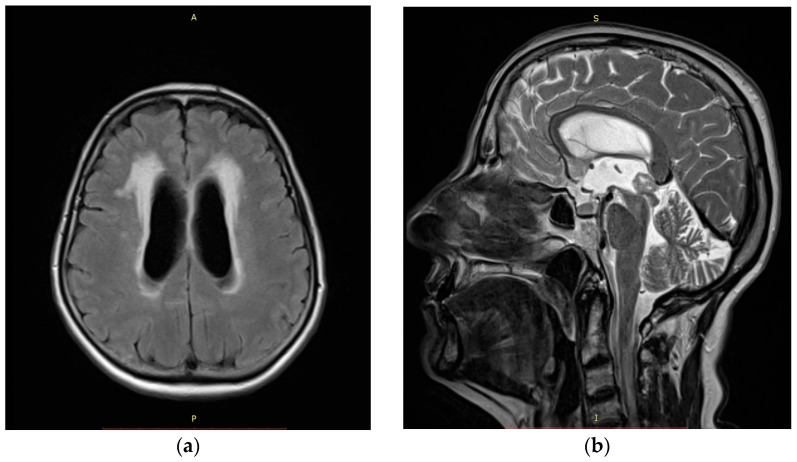
(**a**) Axial plane T2-weighted-fluid-attenuated inversion recovery (FLAIR) MRI scan of patient with pineocytoma and radiological signs of obstructive hydrocephalus included in our study. (**b**) Sagittal plane T2 fast spin echo (FSE) MRI scan in the same patient.

We conducted a retrospective analysis of all patients with intra- and paraventricular brain lesions who underwent neuroendoscopic biopsy between January 2014 and December 2024 at our institution (University Clinical Centre of Vojvodina, Novi Sad, Serbia). The aim of all biopsy techniques was to obtain an adequate tissue sample for subsequent histopathological diagnosis. The primary endpoint of the study was the diagnostic yield of the two biopsy techniques, defined as the proportion of procedures resulting in a conclusive histopathological diagnosis. The secondary endpoints included perioperative and postoperative complications associated with each technique, such as intraventricular hemorrhage, neurological deficits, and other procedure-related adverse events. Due to the small sample size and the retrospective nature of the study, a descriptive analysis was performed. Formal statistical comparisons between groups were not undertaken, as the limited number of patients precluded meaningful inferential statistical analysis. Approval for this study was obtained from the Ethics Committee of the University Clinical Centre of Vojvodina. The patient’s age ranged from 43 to 81 with a mean age of 63.7 and a median of 63.5. A total of 16 patients were included in the study (9 female and 7 male patients). Seven patients presented with confusion and disorientation as major symptoms. Other symptoms that led to radiological diagnostics were headache, gait disturbance, urinary disturbance, and dizziness. Preoperatively, all patients underwent a non-contrast and gadolinium-enhanced MRI scan. Imaging examples of patients included in our study are shown in Figure 1 and Figure 2. Data on the pre- and perioperative clinical course, including postoperative follow-up examinations and radiological findings on postoperative CT and MRI head scans, were obtained retrospectively. Patient data is presented in Table 1.

### 2.2. Neuroendoscopic Technique

All procedures were performed with patients under general anesthesia. Patients were placed in the supine position with the head in 30 degrees of anteflexion. For all procedures, the universal rigid neuroendoscopic system was used (Karl Storz GmbH & Co., Tuttlingen, Germany). In brief, a burr hole was placed at the Kocher’s point, followed by durotomy, corticotomy, and puncture of the lateral ventricle. Following identification of anatomic landmarks of the lateral ventricle, including the foramen of Monro, the fornix, the choroid plexus, the septum pellucidum, and the major venous structures, the wall of the lateral ventricle was examined for possible changes on the surface due to the tumor. In cases of obstructive hydrocephalus, ETV was performed before biopsy.

### 2.3. Biopsy Technique

After insertion of the optics, the endoscope was fixed with the endoscope holder in the direction of the planned biopsy. Under direct endoscopic guidance, the biopsy was performed following initial cauterization of the planned entry site on the surface of the tumor or ventricle wall.

**Figure 3:**
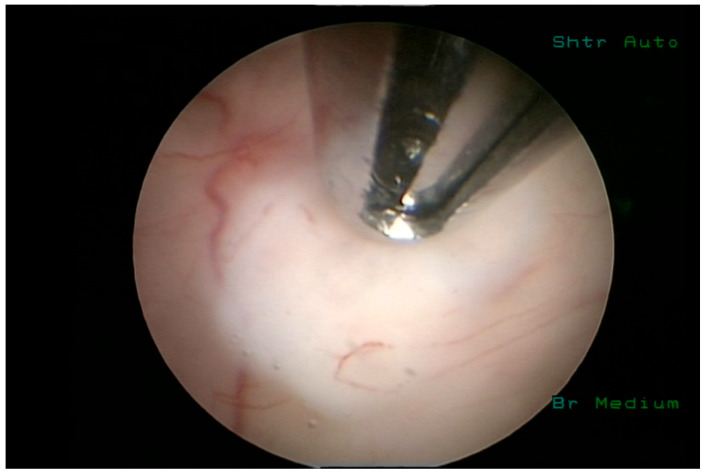
Intraoperative screenshot illustrating moment of insertion of the side-cutting needle into the tumor tissue trough surface of the ventricle.

In the first group of patients, tissue sampling was performed under direct visualization of the lesion from the core of the lesion with a side-cutting needle as illustrated by intraoperative screenshot in Figure 3. After the needle was removed, the entry site was inspected for hemorrhage. The second group of patients underwent a standard biopsy with tissue sampling performed using a cup forceps following a cauterization of biopsy site.

**Figure 4:**
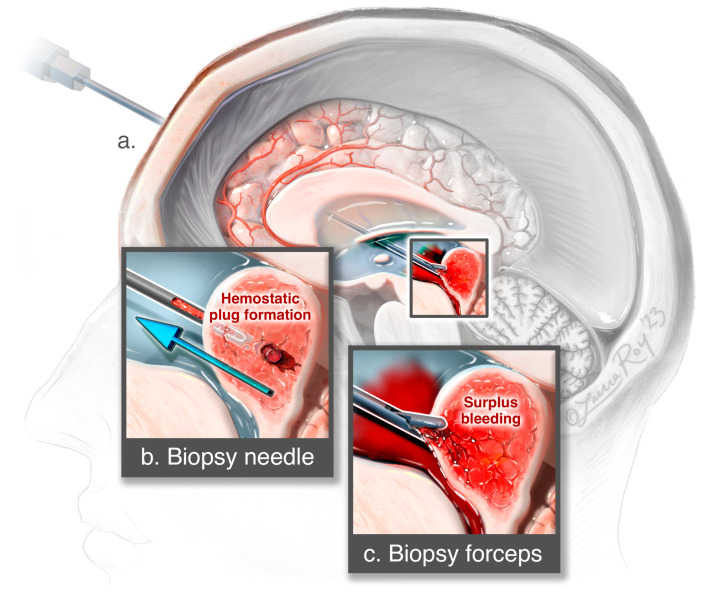
Artists’ depiction of the endoscopy technique. (**a**) Sagittal cross-section illustrates trajectory and placement of endoscope within the ventricular system. (**b**) Inset on the left side illustrates side-cutting needle biopsy technique and formation of the hemostatic plug during the withdrawal of the needle. (**c**) Inset on the right side reveals forceps biopsy and illustrated surplus bleeding from the tumor surface. ©Laura Roy, published with permission.

Figure 4 illustrates both techniques. In total, from 2 to 4 biopsy specimens were taken in each patient.

In both groups, special attention was given to the surface of the ventricle wall and possible bleeding following the biopsy. A final inspection of the ventricle was performed before removing the endoscope.

## 3. Results

A total of 16 patients who underwent a neuroendoscopic biopsy for intracranial lesions were included in this study. No intraoperative complications were noted. The duration of the surgical procedure was in the range of 30 to 107 min with a median time of 61 min. A histopathological diagnosis was obtained in 15 (93.7%) patients. In all procedures, a direct visualization of a pathological lesion was feasible. And in all patients, hemostasis was feasible.

Six (37.5%) patients presented with hydrocephalus at admission and were treated with ETV. ETV was feasible in all procedures using a cauterization of the floor of the third ventricle followed by fenestration and dilatation utilizing a balloon catheter. In one patient (6.25%), isolated lateral left ventricular enlargement was observed, which was caused by compression on the left foramen of Monro due to the tumor localization in the head of the caudate nucleus and was treated with a septum pellucidotomy. In all patients, immediate postoperative course was uneventful. In two patients (12.5%), later clinical course was complicated with a worsening of neurological status due to the persistent hydrocephalus, and they subsequently underwent a shunt procedure.

In all patients, the tumor site could be identified for its prominence into the ventricular system.

Taking into account radiological findings, tumor characteristics, and the surgeon’s experience, two biopsy techniques were applied for obtaining tissue samples. The first group of 11 (68.7%) patients underwent biopsy with a side-cutting needle. The remaining five (31.3%) patients underwent biopsy with cup forceps.

**Figure 5:**
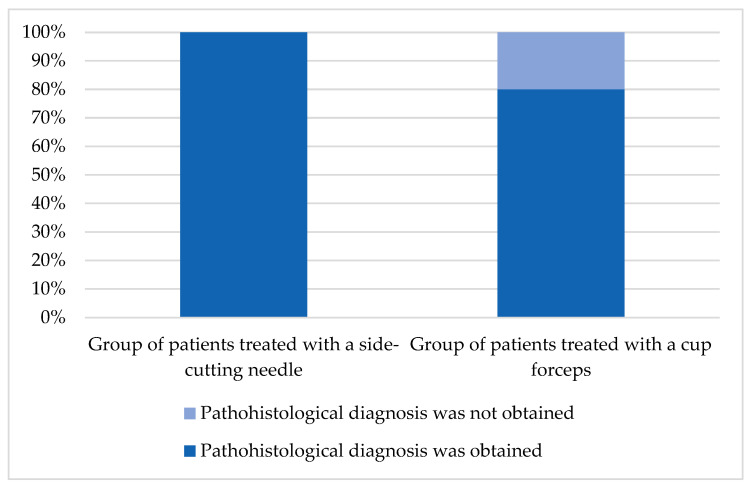
Success rate of pathological histology acquisition in two patient groups.

The histological types of tumors determined with biopsy of the lesion were as follows: six (37.5%) glioblastomas, five (31.2%) pineocytomas, two (12.5%) primary central nervous system lymphomas, one (6.2%) meningioma (WHO grade I), one (6.2%) ependymal tissue, and as mentioned above, in one patient the histological finding was inconclusive. According to the biopsy technique-based groups, the pathological findings were in the first group of patients treated with a side-cutting needle as follows: 4 out of 11 glioblastomas (36.36%), 4/11 pineocytomas (36.36%), 2/11 CNS lymphomas (18,18%) and one out of eleven meningioma (9.09%). In the second group, one out of five was found to be ependymal tissue (20%), 1/5 was pineocytoma (20%), two out of five were glioblastomas (40%), and in one patient pathohistological diagnosis was not established; therefore, histopathological diagnosis was established in all patients who were treated with a side-cutting needle and in 80% of the patients who were treated with cup forceps as shown in Figure 5. The meningioma was eventually treated with surgical resection of the tumor.

Regarding bleeding within the biopsy area, only minor local bleeding following tissue sampling was observed. Hemostasis was obtained with continuous irrigation in all of the cases where minor bleeding was observed. Postoperative CT scans were performed on the following day after surgery and they showed no signs of ventriculorrhagia or intraparenchymal hemorrhage at the endoscope insertion site in all patients, as well as no sign of bleeding at the biopsy site. In the postoperative period, all patients were admitted to the ward.

## 4. Discussion

With rapid technological advances that have taken place over the past and current century, neurosurgery has also experienced substantial progress as well. Regions of the brain that were once surgically inaccessible or accessible with a higher risk of postoperative complications have become less hazardous, with reduced rates of morbidity and mortality [18]. Neuroendoscopic surgery represents a critical milestone in this evolution. Complex intraventricular anatomy and the presence of important structures within this region are no longer excluding criteria for operative management. Instead, neuroendoscopy has enabled safer access to lesions previously considered surgically inaccessible [19,20]. Nowadays, among the most important factors influencing patient outcomes are the surgeon’s skill and experience, accompanied by strategic preoperative planning that prioritizes minimizing the risk for potential complications while achieving a direct therapeutic result.

As noted earlier, intra- and paraventricular tumors encompass a broad histopathological spectrum. Histopathological verification therefore remains essential for determining the appropriate treatment strategy. However, there are no available prospective-randomized trials which confirm the superiority of neuroendoscopic biopsy over stereotactic biopsy [4,13]. There are some undeniable advantages of neuroendoscopy which allow direct visualization of the biopsy site and real-time intraoperative strategy adjustments. Frameless and frame-based stereotactic procedures are based on preoperative radiological findings with an entire operative plan being made prior to the procedure, with a strictly determined entry and target point. This excludes factors such as brain shift due to loss of cerebrospinal fluid or potential shift of lesions while inserting an instrument, as well as the effect of enlargement of ventricles in cases of intraventricular tumors that lead to obstructive hydrocephalus [5]. This can all lead to insufficient sampling, especially in cases of smaller regions, which leads to reoperations or using an alternative trajectory which is associated with greater risk of hemorrhage [21,22].

In neuroendoscopic procedures, even with rigid endoscopes, a minimal movement is allowed, which might be enough to obtain an optimal trajectory for planned biopsy. Even so, the current literature reports the variable feasibility of obtaining reliable diagnostic tissue using neuroendoscopic biopsy. This is most often caused by an insufficient amount of sample collected during biopsy, an inaccurate sampling site, as well as the use of coagulation prior to taking a biopsy sample [5,10,23,24]. This is where the present series and the use of a side-cutting needle demonstrate their value. Considering the fact that cauterization that precedes biopsy is only superficial, deeper tumor tissue where the diagnostic material is obtained remains unaffected, thereby improving the likelihood of acquiring a representative sample for accurate histopathological analysis [13]. Also, in this way, there is a lesser chance of obtaining just the ependymal tissue in cases where pathological lesions are covered with it. We argue that combining the possibility of direct visualization in endoscopic biopsy with the precise instruments for stereotactic biopsy and collecting tissue samples from the intratumoral core rather than the superficial region is the best way of obtaining histopathological diagnosis and minimizing the risk of hemorrhage in a large number of patients.

One of the main advantages of neuroendoscopic procedures remains direct visualization and real-time management of potential intraoperative hemorrhage. Hemorrhage-related complications were found to occur in 3.6–4.8% of stereotactic procedures [25]. Surprisingly, only a few publications have addressed the risk of postoperative bleeding considering the location of the lesions being biopsied. Sato et al. published a retrospective analysis, which concluded that the perioperative hemorrhage was statistically significant, and more often observed in lesions in the basal ganglia compared to other locations [26]. In comparison to stereotactic biopsy, recent studies show that intraoperative hemorrhage occurs in neuroendoscopic procedures in about 3% of the cases [15]. In most cases, it is only minor bleeding that can be managed with warm Ringer’s solution irrigation, which aligns with the findings of our study. Irrigation plays the key role in hemostasis not just for bleeding control but also for visualization of protentional blood vessel lesions, which further enables hemostasis with cauterization if required [15]. Moreover, we argue that using a side-cutting needle additionally plays a role in containing the hemorrhage inside the tumor and therefore decreasing the chance of further hemorrhage. We theorize that the insertion of a side-cutting needle into the depth of the tumor, its withdrawal under the compressive effect of the surrounding tissue and without disruption of the tumor’s superficial layer results in the formation of intratumoral hemostatic plug, thereby reducing the risk of intraventricular hemorrhage. In most cases where extensive intraventricular hemorrhage occurred and could not be contained intraoperatively, patients were treated with external ventricular drainage (EVD) [27]. No patients in our study needed an additional EVD placement, as no hemorrhage-related complications were found at the biopsy site nor were there signs of intraventricular cloths on postoperative CT findings.

One of the biggest advantages of the neuroendoscopic biopsy remains the fact that a simultaneous CSF diversion can be performed in patients which present with obstructive hydrocephalus [9]. Occurrence of hydrocephalus at presentation in patients with intra- and paraventricular lesions depends on the location of the lesion and degree of obstruction. Compression on the pineal and tectal region is more likely to cause obstructive hydrocephalus because of the proximity to the aqueduct, whereas tumors in the proximity to the foramen of Monro and in the region of the basal ganglia are more likely to cause dilation of one or both lateral ventricles. As it was shown in our results, one patient with glioblastoma in the region of the basal ganglia presented with unilateral ventricular enlargement and was treated with septum pellucidotomy, which was shown to be a successful strategy to deal with this type of hydrocephalus in the available literature [9,16]. Still, in the majority of cases, ETV remains the best option for management of obstructive hydrocephalus due to compression on the aqueduct with the procedure’s efficacy reported to be 70% to 95% with a long-term success rate of 83% [16,28,29]. In our study, a total of six patients underwent ETV with two of them needing an additional shunt procedure in the postoperative period within ten days after the initial surgery. We argue that this is due to large tumor masses as well as insufficient ETV outflow. The literature findings support this theory while also differing in perioperative versus late ETV failure [30]. In the first group, failure happens because the new stoma ceases to function due to inefficient size or there being another membrane underneath, whereas the other group of patients experience progression of the hydrocephalus due to reclosure of the stoma due to scarring or gliosis [31,32]. In those cases, shunt procedures still remain the best option for hydrocephalus treatment.

Although using a side-cutting needle has its many advantages as we have shown in our study, there is still a place for using a standard procedure with cup forceps in cases where pathological lesions are superficial—keeping in mind that they are not located subependymally, where dimensions of the tumor do not allow a side-cutting needle to be inserted in it—and in the case of intratumoral hemorrhage [33].

## 5. Conclusions

In summary, the present study shows that neuroendoscopic biopsy has become a standard practice in the treatment of para- and intraventricular lesions because of its superiority to stereotactic procedures in the real-time management of events. Regardless of its bleeding control or necessity of intraoperative adjustment to prior planning, direct visualization, its somewhat flexible nature, and its ability to react in real time make this procedure valuable because it gives surgeons better control over the situation. Nevertheless, a constant need for improvement leads to innovations such as a neuroendoscopic procedure combined with a side-cutting needle from stereotactic biopsies described in this paper.

Although the focus of this study was primarily placed on the technique combining neuroendoscopic and stereotactic approaches, it is important to emphasize that both biopsy techniques have a well-established role in clinical practice. While the side-cutting needle demonstrated several advantages in terms of tissue representativeness and bleeding control in selected cases, the conventional cup forceps technique remains a valuable option for superficially located lesions that are not subependymal, for tumors whose dimensions do not allow safe insertion of a side-cutting needle, and in cases of intratumoral hemorrhage. In our series, the side-cutting biopsy needle demonstrated a higher diagnostic yield compared to the conventional cup forceps technique (100% vs. 80%), while enabling more adequate tissue sampling, especially with subependymal lesions, without bleeding or with only minimal hemorrhagic events.

Optimal patient outcomes are therefore best achieved through careful patient selection and an individualized surgical strategy, aiming to maximize diagnostic yield while minimizing the risk of hemorrhagic complications.

## Figures and Tables

**Table 1:** Patient collective and symptoms.

Patient	Age	Sex	Symptoms	Localization of Pathology	Obstructive Hydrocephalus	Biopsy Technique	Combined Procedure	Complications
Patient 1	61	M	Urinary and gait disturbance	Third ventricle	Yes	Side-cutting Needle	ETV	Persistent Hydrocephalus
Patient 2	72	F	Confusion, urinary disturbance	Pineal region	Yes	Side-cutting Needle	ETV	No
Patient 3	43	M	Vertigo, gait disturbance	Third ventricle	Yes	Side-cutting Needle	ETV	Persistent Hydrocephalus
Patient 4	54	F	Headache, confusion	Left thalamus	Yes	Side-cutting Needle	ETV, pellucidotomy	No
Patient 5	78	M	Confusion, disorientation	Right thalamus	No	Side-cutting Needle	No	No
Patient 6	63	F	Vertigo, vomitus	Right thalamus	No	Side-cutting Needle	No	No
Patient 7	81	F	Confusion, disorientation	Right thalamus	No	Side-cutting Needle	No	No
Patient 8	53	M	Headache	Third ventricle	Yes	Side-cutting Needle	ETV	No
Patient 9	72	F	Confusion, gait disturbance	Left thalamus	No	Side-cutting Needle	No	No
Patient 10	69	F	Confusion, disorientation	Pineal region	Yes	Side-cutting Needle	ETV	No
Patient 11	64	F	Confusion, disorientation	Pineal region	Yes	Side-cutting Needle	ETV	No
Patient 12	64	M	Confusion, disorientation	Left thalamus	Yes	Cup Forceps	ETV	No
Patient 13	50	F	Headache	Left thalamus	No	Cup Forceps	No	No
Patient 14	61	F	Confusion, disorientation	Right thalamus	No	Cup Forceps	No	No
Patient 15	74	M	Confusion, disorientation	Right thalamus	Yes	Cup Forceps	ETV	No
Patient 16	51	M	Vertigo, vomitus	Left thalamus	Yes	Cup Forceps	ETV	No

F, female; M, male; ETV, endoscopic third ventriculostomy.

## Data Availability

All data generated or analyzed during this study are included in this article. Further enquiries can be directed to the corresponding author.

## Abbreviations

The following abbreviations are used in this manuscript:

ETV	Endoscopic third ventriculostomy
EDV	External ventricular drainage
